# Clinical course and complications following diagnostic bronchoalveolar lavage in critically ill mechanically ventilated patients

**DOI:** 10.1186/s12890-015-0104-1

**Published:** 2015-09-29

**Authors:** R M Schnabel, K van der Velden, A Osinski, G Rohde, P M H J Roekaerts, D C J J Bergmans

**Affiliations:** Department of Intensive Care Medicine, Maastricht University Medical Centre+, P. Debyelaan 25, PO Box 5800, 6202 AZ Maastricht, The Netherlands; Department of Respiratory Medicine, Maastricht University Medical Centre+, Maastricht, The Netherlands

**Keywords:** Bronchoalveolar lavage, Pneumonia, Safety, Complications, Critical care

## Abstract

**Background:**

Flexible, fibreoptic bronchoscopy (FFB) and bronchoalveolar lavage (BAL) have been used for diagnostic purposes in critically ill ventilated patients. The additional diagnostic value compared to tracheal aspirations in ventilator-associated pneumonia (VAP) has been questioned. Nevertheless, BAL can provide extra information for the differential diagnosis of respiratory disease and good antibiotic stewardship. These benefits should outweigh potential hazards caused by the invasiveness of this diagnostic technique. The focus of the present study was on the clinical course and complications of patients following BAL procedures up to 24 h.

**Methods:**

Hundred sixty-four FFB guided BAL procedures for suspected pneumonia were analysed in an observational study. The clinical course of patients was monitored by respiratory and haemodynamic data before BAL, 1 and 24 h after BAL. Complications were defined and registered. Factors associated with complications were analysed by logistic regression.

**Results:**

Clinical course: a decrease in average pO_2_/FiO_2_ ratio 1 h after BAL from 29 kPa (218 mmHg) to 25 kPa (189 mmHg) (p < 0.05) was observed which fully recovered within 24 h. Respiratory complications: the incidence of procedure related hypo-oxygenation (SaO_2_ ≤ 88 %) and/or bronchospasm was 9 %; a decrease of >25 % PaO_2_/FiO_2_ ratio 1 h after BAL was found in 29 % of patients; no bleeding or pneumothorax were registered. Haemodynamic complications: there were no cases of hypertension and cardiac rhythm disturbances; haemodynamic instability within the first 24 h after BAL was recorded in 22 %; this was correlated with a cardiovascular diagnosis at admission (OR 2.9; 95 % CI 1.2 - 6.7) and the presence of cardiovascular co-morbidity (OR 3.5; 95 % CI 1.5 – 8.3). The incidence of bacteraemia was 7 %. There was no case of procedure related death.

**Discussion:**

Frequently occurring haemodynamic and respiratory instability but no cases of cardiac rhythm disturbances, bleeding, pneumothorax or procedure related death were attributable to diagnostic FFB and BAL. The procedures should be conducted under careful supervision by experienced physicians. Only a randomized controlled trial that compares diagnostic FFB and BAL with a non-invasive strategy could ultimately establish the safety profile and clinical utility of these procedures in critically ill ventilated patients.

## Background

Flexible, fibreoptic bronchoscopy (FFB) and bronchoalveolar lavage (BAL) have been established for diagnostic purposes in critically ill ventilated patients in intensive care units (ICUs). The technique is applicable at the bedside and therefore makes potentially hazardous patient transfers outside the ICU unnecessary. With regard to the diagnosis of ventilator-associated pneumonia (VAP) there is no consensus in the literature and between clinicians. Bronchoscopic BAL from the presumed site of infection with cytological analysis and quantitative microbiological culture of the lavage fluid is thought by many to be a specific diagnostic approach to identify patients with a true infection of the lungs and to enable tailored antibiotic therapy. The most cited benefit of BAL is the prevention of incorrect use of antibiotics. The administration of unnecessary antibiotics is associated with risks of toxicity and development of multi-resistance [1–3]. Others question the use of BAL because several investigations have shown similar outcome and overall use of antibiotics when clinical diagnostic criteria added by non-quantitative culture results from tracheal aspirations were used in the diagnosis of VAP [4, 5]. However, there are other benefits associated with the performance of BALs. Direct visualisation of the airway by FFB delivers additional clinical information and guides the selection of lung areas where specimens are taken [6, 7]. Moreover, the information provided by BAL analysis is more comprehensive than from tracheal aspirates as it not only can benefit the diagnosis of bacterial VAP [8] but can also support the diagnosis of alternative infectious causes [9] or other respiratory diseases [10].

In order to evaluate FFB and BAL as a diagnostic tool in ICU patients, it is necessary to balance benefits with potential hazards caused by the invasiveness of the technique. The focus of the present study is on the clinical tolerance of FFB and BAL. The clinical course and complications of critically ill mechanically ventilated patients after a diagnostic FFB and BAL were investigated. Clinical data were collected before BAL, 1 and 24 h after BAL. The frequency of haemodynamic, respiratory and procedure related complications were registered. This information can contribute to the discussion whether or not utility and benefits of the more invasive diagnostic approach outweigh its potential risks.

## Methods

The study was conducted in the Maastricht University Medical Centre+, a tertiary-care, university hospital in the Netherlands with 1,700 ICU admissions per year. Adult critically ill, mechanically ventilated patients with a clinical suspicion of infectious pneumonia who underwent a diagnostic FFB and BAL were included in the analysis. Exclusion criteria for the procedure were thrombocytopenia (<40,000/μL) and other coagulation abnormalities. Only clinical data were used in the study. The data were collected retrospectively in 43 cases and prospectively in 121 cases in the period from 2011 to 2014. Patients consented to the use of clinical data for scientific analysis according to the admission regulations of the hospital if they did not opt out. This is approved by the local ethics commission (Medisch Ethische Toetsingscommissie azM).

Senior registrars or consultant pulmonologists conducted all BAL procedures. The fraction of inspired oxygen (FiO_2_) was increased to 1.0 for 5 to 15 min prior to and during FFB and BAL procedures. Patients were *anaesthetised* by an experienced intensive care physician, present during the whole procedure, with either propofol or midazolam and fentanyl continuous infusion and as required rocuronium for muscle relaxation. Patients had tracheal tubes with an inner diameter varying from 7.5 to 8.5 mm or a Portex® tracheal cannula with an inner diameter of 8 mm in place. A flexible, fibreoptic bronchoscope (Pentax® FB-15H/FB-15X, Pentax Medicals, Tokyo, Japan) with a calibre of 4.9 mm was introduced and ‘wedged’ into the affected segmental or subsegmental bronchus. Before performing BAL, a quick inspection of all the major airways in both lungs was carried out. Sterile saline (0.9 % sodium chloride at room temperature) was instilled. An initial aliquot of 20 mL and subsequent four aliquots of 50 mL were immediately aspirated and recovered. After completing the BAL, the bronchoscope was removed, and the patient was monitored closely by continuous pulse oximetry and arterial blood gases while the FiO_2_ was then gradually decreased. BAL samples were transported to the laboratory within 15 min after collection and processed immediately upon arrival. Information about the quality of BAL sampling was collected. The BAL fluid was regarded non-representative if the volume of recovery was <20 mL, the total cell count was < 60.000/mL, the presence of squamous epithelial cells was >1 %, the presence of bronchial epithelial cells was >5 % or in the presence of extensive amounts of debris and damaged cells. Further BAL workup in the laboratory included: a differential cell count, microscopic investigation of a Gram-stained preparation, quantitative bacterial and fungal culture. BAL fluid analysis was considered to support the diagnosis of VAP when Memphis Consensus Conference criteria were met. These criteria include: ≥2 % BAL fluid cells containing intracellular organisms, and/or identification of a bacterial micro-organism in the BAL fluid in a concentration of ≥ 10^4^ colony forming units/ml (cfu/mL) [11, 12]. Demographic characteristics and Acute Physiology and Chronic Health Evaluation (APACHE-II) scores were recorded on admission. All diagnoses apart from the principal reason for admission to the ICU were regarded as co-morbidities: 1. cardiovascular: severe ischemic heart disease, NYHA IV heart failure, disabling peripheral vascular disease; 2. respiratory: disabling chronic pulmonary disease, COPD Gold III and IV, continuous additional oxygen therapy; 3. chronic renal failure: chronic dialysis, serum creatinine level chronically > 177 μmol/L; 4. immunocompromised: disease and drug effects, prednisone equivalent > 7.5 mg/day; 5. active malignancy; 6. neurologic impairment: cerebral haemorrhage, meningitis, encephalitis, severe paresis; 7. chronic coagulation disorder; 8. chronic hepatic failure: hepatic encephalopathy, Child C cirrhosis, portal hypertension. Patients were classified as “severe sepsis” following the Surviving Sepsis Campaign criteria [13]. Clinical parameters were collected at baseline, at 1 h and 24 h after the BAL procedure. Blood cultures were obtained before and 24 h after the procedure, chest X-rays were performed within 24 h after BAL and sputum cultures were taken prior to the procedure. In general, the results of Gram staining, differential cell count and percentage of intracellular organisms of samples obtained by BAL were available within 2 h after bronchoscopy, preliminary culture results after 1 day, and definite culture results after 4 days. When BAL fluid analysis rejected the diagnosis of bacterial pneumonia, additional steps followed to determine an alternative diagnosis. Empirical antibiotic therapy was directed towards pathogens isolated from clinical and surveillance cultures of sputum cultures, and patients with VAP were treated with antibiotics for 8 to 14 days according to causative microorganisms. In case of clinical suspicion of pneumonia, antibiotic therapy was initiated after BAL had been performed. When a patient was receiving antibiotics for more than 48 h for another unrelated infection or for prophylaxis on the day that the criteria for a clinical suspicion of pneumonia were met, antibiotics were not changed until the BAL procedure.

The clinical course of the patients was monitored for 24 h after diagnostic FFB and BAL. PaO_2_/FiO_2_ ratios, positive end-expiratory pressure (PEEP) levels, fractions of oxygen (FiO_2_), ph, PaCo_2_, mean arterial pressure (MAP), heart rate and dosages of norepinephrine and dobutamine were assessed. Clinical complications of BAL were defined as complications occurring during the BAL procedure and up to 24 h after BAL. *1. Respiratory complications:* hypo-oxygenation (SaO_2_ ≤ 88 %) during BAL and/or bronchospasm; a decrease in PaO_2_/FiO_2_ ratio >25 % compared to baseline; bleeding defined as haemoptysis requiring medical intervention and/or interruption of the procedure; pneumothorax*. 2. Haemodynamic complications:* hypertension defined as an increase of MAP >25 % baseline; cardiac rhythm disturbances defined as newly developed pathologic rhythm (atrial fibrillation, ventricular tachycardia, ventricular fibrillation, asystole) or bradycardia or rapid atrial fibrillation with decrease in MAP; haemodynamic instability defined as a decrease of MAP < 55 mmHg at any time and/or necessity to initiate norepinephrine > 0.15 μg/kg/min or dubutamine >5 μg/kg/min and/or the necessity to more than double the dose of norepinephrine or dobutamine. *3. Bacteraemia:* positive blood cultures following BAL. *4. Death:* procedure related death within 24 h. Possible correlations of demographic and clinical items (age, gender, APACHE II, SOFA, diagnosis at admission, severe sepsis, co-morbidities) with a respiratory and hemodynamic complication following BAL were analysed by multiple logistic regression. Odds ratios with 95 % confidence interval were calculated.

Data were analysed using the SPSS version 20 statistical package for MS Windows. Quantitative variables were expressed as means with standard deviations (SD). Qualitative variables were reported with the percentage distribution of each of the categories. Fisher’s Exact Test and Chi Square Test were used for the analyses of categorical variables and ANOVA analysis and Kruskal-Wallis test for numerical variables. Linear mixed models analysis was performed in all patients, excluding consecutive BALs in the same patients who underwent more than one BAL.

## Results

A total of 164 datasets of FFB and BAL procedures were recorded during the study period.

Patient demographics are shown in Table 1. In 68 patients (41 %) analysis of BAL fluid supported the diagnosis bacterial pneumonia. The most frequently acquired micro-organisms were *Pseudomonas aeruginosa* (12), *Staphylococcus aureus* (5) and *Escherichia coli* (5). In retrospection 92 % of all BAL positive patients received adequate empiric antibiotic treatment. Ninety-six BAL fluid analyses were classified as negative. However, in 68 % of these an alternative diagnosis could be established in the following diagnostic process [Table 2]. In 26 patients there was a significant presence of viruses in the BAL fluid. In seven patients *Pneumocystis jiroveci* could be detected. Further diagnostic workup including computed tomography, lung biopsy, ultrasound examinations and advanced laboratory tests revealed a broad spectrum of alternative diagnoses in the putative ventilator-associated pneumonia patient not meeting lavage-based diagnostic criteria.

**Table 1:** Patient demographics

Age (years)		61	±14
Gender	male	116	71 %
	female	48	29 %
APACHE II		21	±9
SOFA (at time of BAL)		7	±4
Parameters (at time of BAL)	pH <7.25	10	6 %
	PaCO_2_ ≥ 7.5kPa (56 mmHg)	9	5 %
	PaO_2_ ≤ 8kPa (60 mmHg)	10	6 %
	PaO2/FiO2 ratio ≤ 13kPa (100 mmHg)	12	7 %
Diagnosis upon admission	respiratory	64	38 %
	cardiovascular	37	22 %
	haematological	19	11 %
	gastrointestinal	16	10 %
	trauma/orthopaedic	10	6 %
	neurological	12	7 %
	urogenital	3	2 %
	other	7	4 %
Severe Sepsis at admission		52	32 %
Co-morbidity	none	53	33 %
	one item	72	44 %
	two items	35	21 %
	≥ three items	4	2 %
Co-morbidity	cardiovascular	34	21 %
	respiratory	12	7 %
	chronic renal failure	8	5 %
	immunocompromised	47	29 %
	active malignancy	32	20 %
	neurologic impairment	17	10 %
	coagulation disorder	3	2 %
	chronic hepatic failure	2	1 %
BAL positive		68	41 %
ICU mortality		80	49 %
In-hospital mortality		90	55 %
Cause of death	cardiovascular	13	15 %
	persistent respiratory failure	24	27 %
	neurological impairment	9	10 %
	multi organ failure	10	11 %
	active malignancy	28	31 %
	other	4	4 %
	unknown	2	2 %

Results are given either as number, percentages or mean ± standard deviation

**Table 2:** Alternative diagnosis in patients with negative bacterial growth BAL result

BAL negative (*n* = 96)	
Viral	26
*Pneumocystis jirovecii*	7
Fungal	6
Heart failure	4
Bronchiolitis obliterans organizing pneumonia	3
Usual interstitial pneumonia	1
All trans retinoic acid (ATRA) syndrome	1
Pulmonary embolism; obstructive shock	2
Endocarditis	1
Abdominal sepsis with ARDS	4
Necrotizing pancreatitis	2
Abdominal ischaemia	1
Urinary tract sepsis with ARDS	2
Ovarian cancer with pulmonary metastases	1
Systemic lupus erythematosus	1
Toxic epidermal necrolysis	1
Postanoxic encephalopathy with multiple infarction	1
Myasthenia gravis	1
None established	31

The clinical course of patients following diagnostic FFB and BAL is illustrated in Table 3. Baseline data before BAL were compared with data after 1 h and 24 h. The average PaO_2_/FiO_2_ ratio declined from 29 kPa (218 mmHg) to 25 kPa (189 mmHg) (*p* < 0.05) after 1 h but had reached baseline again after 24 h. The average PEEP level of 9.4 cmH_2_O was not significantly increased after 1 h and kept constant after 24 h. After 1 h, PEEP level was increased in 7 % of patients and in 15 % after 24 h. The average FiO_2_ level of 52 % was not significantly altered after 1 h and had decreased to 43 % (*p* < 0.0001) 24 h after BAL. One hour after BAL, 27 % of patients had an increased FiO_2_ of ≥ 10 % compared to baseline; after 24 h 8 % of patients had an increased FiO_2_ of ≥ 10 % compared to baseline. The pH and paCO_2_ were unchanged after 1 and 24 h compared to baseline. The averages of haemodynamic parameters (MAP, heart rate, number of patients receiving norepinephrine and/or dobutamine and dose) were not significantly different after 1 and 24 h from baseline.

**Table 3:** Results - clinical course 1 h and 24 h after BAL procedure

	Before BAL	1 h after BAL	24 h after BAL
PaO_2_/FiO_2_ [kPa]	29 (CI 25–32)	25 (CI 23–29) p < 0.05	31 (CI 27–33)
PaO_2_/FiO_2_ [mmHg]	218 (CI 188–240)	189 (CI 173–218)	233 (CI 203–248)
PaO_2_/FiO_2_ decreased >25 % baseline [% of patients]		29 %	14 %
PEEP mean [cmH_2_O]	9.4 (CI 9.1–9.8)	9.6 (CI 9.2–9.9)	9.6 (CI 9.2–10)
PEEP increased		7 %	15 %
FiO_2_ mean [%]	52 (CI 49–55)	54 (CI 51–57)	43 (CI 41–46) *p* < 0.0001
FiO_2_ increased ≥ 10 % baseline [% of patients]		27 %	8 %
pH mean	7.41 (CI 7.40–7.42)	7.40 (CI 7.39–7.41)	7.41 (CI 7.40–7.42)
pCO_2_ mean [kPa]	5.2 (CI 5.0–5.4)	5.4 (CI 5.2–5.6)	5.2 (CI 5.0–5.4)
pCO_2_ mean [mmHg]	39 (CI 38–41)	41 (CI 39–42)	39 (CI 38–41)
MAP mean [mmHg]	84 (CI 81–86)	79 (CI 76–81)	83 (CI 81–86)
Heart rate mean [beats/min]	96 (CI 93–100)	94 (CI 93–99)	91 (CI 88–94)
Norepinephrine [% of patients]	58 %	57 %	62 %
Norepinephrine median	0.125	0.109	0.125
1. quartile/3. Quartile [μg/kg/min]	0.050/0.201	0.075/0.202	0.068/0.219
Dobutamine [% of patients]	3 %	3 %	3 %
Dobutamine median	5	5	3
1. quartile/3. Quartile [μg/kg/min]	3/5	2/5	3/5
Hemodynamic instability^a^[% of patients]		5 %	7 %
Hemodynamic instability^a^ at any time within 24 h [% of patients]			22 %

*CI* 95 % confidence interval, *MAP* mean arterial pressure, *PEEP* positive end-expiratory pressure, *FiO*_*2*_ fraction of inspired oxygen, *PaO*_*2*_ arterial partial oxygen pressure, *SOFA* sequential organ failure assessment

^a^decrease of mean arterial pressure (MAP) < 55 mmHg and/or necessity to initiate norepinephrine > 0.15 μg/kg/min / dubutamine >5 μg/kg/min and/or the necessity to more than double the dose of norepinephrine or dobutamine

**Fig. 1:**
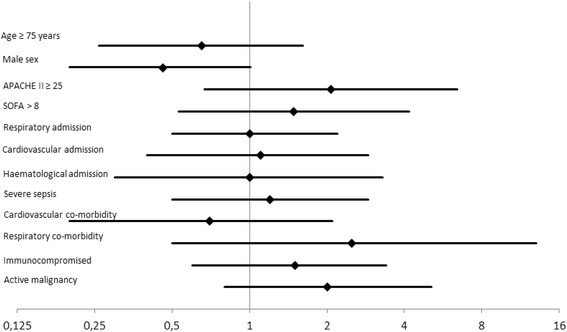
Results – Odds ratios and 95 % confidence interval for respiratory complications^1^ of BAL. ^1^ Decrease of the PaO_2_/FiO_2_ > 25 % compared to baseline within 1 h after BAL

**Fig. 2:**
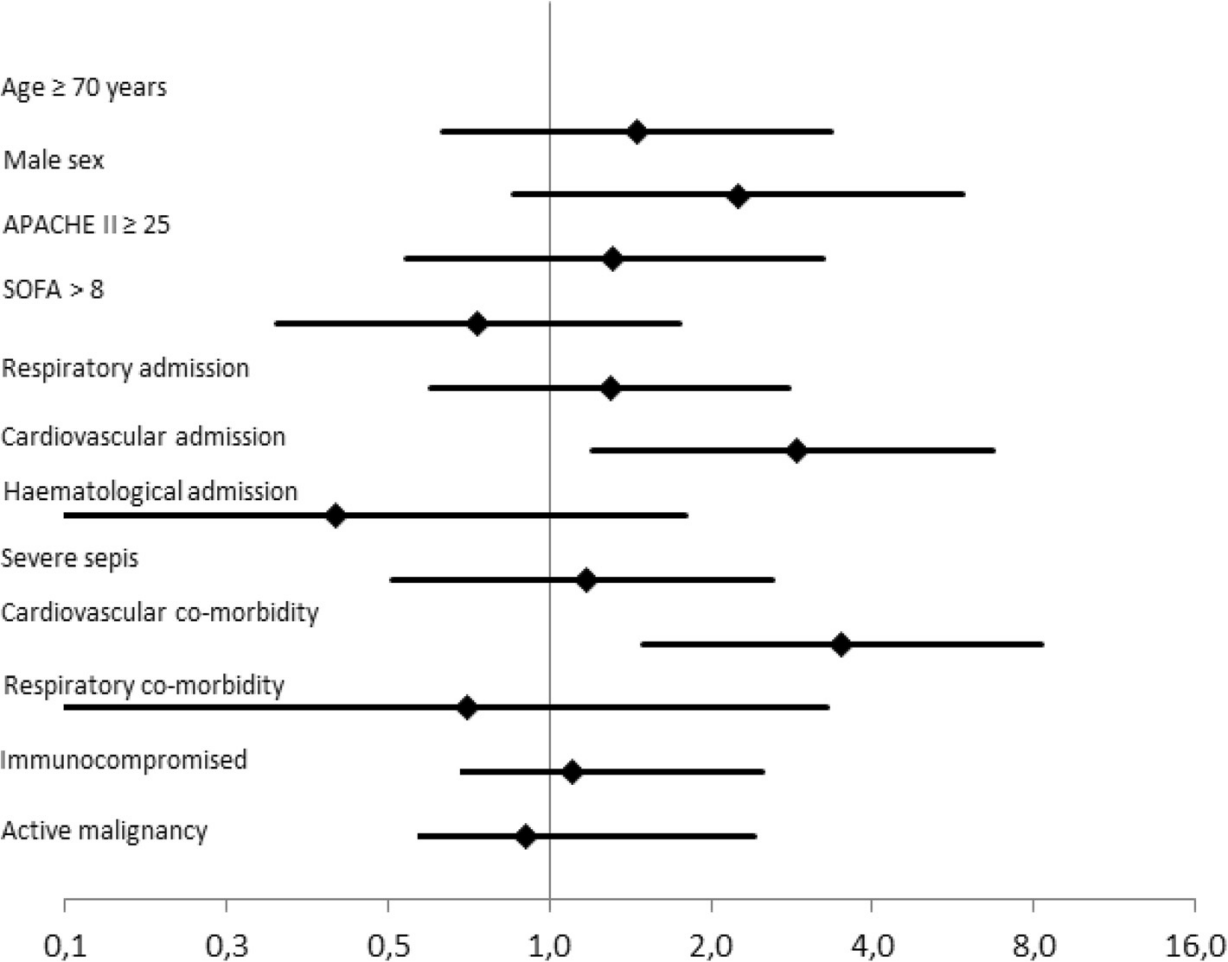
Results – Odds ratios with 95 % confidence interval for hemodynamic complications ^1^ of BAL. A cardiovascular diagnosis upon admission and cardiovascular co-morbidity are associated with significantly more hemodynamic complications. ^1^Decrease of mean arterial pressure (MAP) < 55 mmHg at any time and/or necessity to initiate norepinephrine > 0.15 μg/kg/min / dubutamine >5 μg/kg/min and/or the necessity to more than double the dose of norepinephrine or dobutamine

The following complications of diagnostic FFB and BAL were registered. *1. Respiratory complications*: hypo-oxygenation (SaO_2_ ≤ 88 %) during BAL and/or bronchospasm was documented in 9 % of patients; a decrease of >25 % PaO_2_/FiO_2_ ratio compared to baseline was registered in 29 % of patients 1 h after BAL; in half of these patients a persisting decrease of >25 % PaO_2_/FiO_2_ ratio was found 24 h after BAL; no cases of clinically significant bleeding requiring interruption of the procedure or treatment were reported. Further analysis could not establish a significant correlation of respiratory complications with demographic or clinic items [Fig. 1]. *2. Haemodynamic complications:* no patients had hypertension or cardiac rhythm disturbances during the FFB and BAL procedure; hemodynamic instability (as defined in the methods section) was found in 5 % of patients 1 h after BAL and 7 % of patients 24 h after BAL. However, 22 % of patients had hemodynamic instability at any time during the first 24 h after BAL. This was correlated with a cardiovascular diagnosis at admission (OR 2.9; 95 % CI 1.2 - 6.7) and the presence of cardiovascular co-morbidity (OR 3.5; 95 % CI 1.5 - 8.3) [Fig. 2]. Multiple logistic regression revealed that the correlation was independent from age and gender (OR 2.3; 95 % CI 1 – 5.6 / OR 2.8; 95 % CI 1.1 – 6.7) and the APACHE II score at admission (OR 3.5; 95 % CI 1.5 – 8.5 / OR 3.5; 95 % CI 1.4 – 8.6) *3. Bacteraemia:* newly positive blood cultures were observed in 7 % of patients. *4. Death:* no case of procedure related death was reported.

## Discussion

The present observational study monitored the 24 h clinical course and complications following diagnostic FFB and BAL in critically ill ventilated patients. Patients’ characteristics illustrate the severity of illness before BAL procedures. The high percentage of patients with active malignancy, immunosuppression and neurologic impairment can be explained by the function of the hospital as referral centre for haematology, oncology, immunologic diseases and neurosurgery.

Following diagnostic FFB and BAL there were no cases of cardiac rhythm disturbances, bleeding, pneumothorax or procedure related death during this study. Nevertheless, haemodynamic (22 %) and respiratory (29 %) instability according to the applied definitions were frequently recorded. Due to the complexity of human nature, physiology and pathophysiologic mechanisms of diseases, there might be no simple and consistent definition of respiratory and haemodynamic instability. It has been notoriously difficult to define cut-off variables with regard to vital parameters in clinical trials and practice [14–16]. A single vital parameter or even a set of absolute values of vital parameters will rarely suffice. For example in patients with preserved or abolished autoregulation the same absolute value of MAP can have different effects on the blood flow and vital functions. Hemodynamic and respiratory variables in critical care and ventilated patients markedly interact. MAP and heart rate should be related to dosages of inotropic and vasoactive medication; partial pressure of oxygen should always be correlated with the applied fraction of oxygen and PEEP level. Relative changes of vital parameters with regard to baseline might provide a better assessment of the impact of invasive diagnostic techniques. The definitions for respiratory and hemodynamic complications in the present study were eventually based on pathophysiologic reasoning, earlier publications of bronchoscopy associated complications [17, 18] and literature [19]. Haemodynamic instability in the study population was associated with a cardiovascular diagnosis at admission or presence of cardiovascular co-morbidity reflecting an expected causal relationship. Interestingly these two risk factors were independent from age, gender and APACHE II score. Haemodynamic changes could have been influenced by cardiovascular effects of the applied anaesthetic drugs to facilitate the procedure, as judged by the supervising physician. Hypo-oxygenation (SaO_2_ ≤ 88 %) during BAL and/or bronchospasm occurred in 9 % of patients and was always manageable and did not necessitate terminating the procedure. Nevertheless, 1 h after BAL there was a significant decrease in the average PaO_2_/FiO_2_ ratio which fully recovered to baseline after 24 h. Average PEEP and FiO_2_ levels remained constant. After 24 h more patients had PEEP adjustment than 1 h after BAL explaining the decrease of the average FiO_2_ from 52 to 43 %.

Altogether, haemodynamic and respiratory instability could be attributed to FFB and BAL in a substantial number of patients. This is in line with previous studies concerning the safety of bronchoscopic diagnostic techniques in critical care patients. Table 4 provides an overview of the literature [17, 18, 20–25]. The studies differ in patient population, setting, applied diagnostic technique and length of observation. All authors described a certain impact of diagnostic FFB on respiratory mechanics, respiratory and hemodynamic parameters.

**Table 4:** Results of studies with regard to bronchoscopically guided diagnostic techniques in ICU patients

Reference	Year	Patients	Setting	Results
Trouillet et al. [20]	1990	*N* = 107	acutely ill ventilated patients; bronchial brush	26 % of patients (*p* < 0.01) with a drop in PaO_2_; modest alterations in mean systolic blood pressure related to sedation; six patients developed arrhythmias during the procedure
Montravers et al. [21]	1993	*N* = 30	mechanically ventilated patients; BAL	mild haemodynamic variations but a marked decrease in PaO_2_ during bronchoscopy with a maximum at the end of the procedure that not fully recovered up to 2 h thereafter
Steinberg et al. [17]	1993	*N* = 110	ARDS patients; BAL	21 patients with a moderate to severe drop in PaO_2_/FiO_2_; four patients with a decrease of mean arterial pressure < 60 mmHg; six patients developed arrhythmias during the procedure; one pneumothorax; two cases of bacteraemia within 24 h
Turner et al. [25]	1994	*N* = 107	intensive care patients; bronchial brush	study describes therapeutic and diagnostic fiberoptic bronchoscopies; 23 bronchial brush procedures included; low rate of complications (4.1 %); none with long-term sequelae
Bauer et al. [22]	2001	*N* = 37	mechanically ventilated patients; BAL	patients showed a lower PaO_2_/FiO_2_ ratio and higher MAP after BAL up to 24 h
Chou et al. [23]	2009	*N* = 56	mechanically ventilated patients; BAL	alterations in respiratory mechanics more pronounced by BAL in patients with intrinsic positive end-expiratory pressure before the procedure was started
Estella [24]	2010	*N* = 50	Mechanically ventilated patients; BAL	respiratory mechanics during BAL procedures showed significant decrease in lung compliance and increase in resistance that only reached baseline after 90 min to 3 h
Estella [18]	2012	*N* = 148	ICU patients; BAL	desaturations <90 % (7 %), supraventricular tachycardia (4 %) and slight bronchial bleeding (2 %)

To warrant a high procedural safety level of FFB and BAL in ICU patients, a structured approach is mandatory [6]. An experienced bronchoscopist should concentrate on the procedure while another physician surveys the patient, regulates analgosedation and adjusts the support of vital functions as necessary. The benefits of a BAL should be weighed against the potential additional harm for the patient secondary to the invasiveness of the procedure and the additional costs as compared to less invasive diagnostic measures. There is ongoing discussion on the pros and cons of BAL in the diagnosis of VAP. In this study, the majority of patients had a substantial benefit from the BAL. In 41 % of patients the diagnosis of bacterial pneumonia was confirmed by BAL fluid analysis. In a further 24 % of patients a viral or fungal infection or *Pneumocystis jiroveci* pneumonia could be diagnosed. When no infectious cause in the BAL fluid analysis was detected, the medical team was urged to look for non-infectious diagnoses without any delay and to adjust the therapy. This practice could beneficially affect the patients’ outcome and at the same time reduce unnecessary antibiotic use. The latter becomes increasingly important with the rapid emergence and dissemination of multi-drug resistant microorganisms particularly in the ICU environment worldwide [26]. Antibiotic stewardship programs (ASPs) were developed to address the problem and ensure a responsible use of antimicrobial drugs [27, 28]. The diagnostic BAL can be implemented in an ASP and deliver important information to confirm an infectious disease, to optimize antimicrobial treatment (e.g. in the presence of *Pneumocystis jiroveci*), to de-escalate and narrow antibiotic therapy once the responsible pathogen is known and to stop antibiotic therapy in patients unlikely to have infections [27, 29].

Limitations of the study are its observational design of FFB and BAL as standard of care in a single centre without a randomized control group. Part of the included cases were retrospectively collected. In the assessment of a long term clinical course it might be difficult to differentiate the natural course of the underlying disease from the impact of the bronchoscopic intervention. Evidently, FFB and BAL procedures should not be performed in patients in a deplorable clinical condition. Unfortunately, the present study does not allow to define clear-cut clinical criteria for withholding FFB and BAL because the limited number of patients with severe acidosis (pH < 7.25; *n* = 10), hypercapnia (PaCo2 ≥ 7.5kPa/56 mmHg; *n* = 9) and hypoxia (PaO2 ≤ 8kPa/60 mmHg; *n* = 10 or PaO2/FiO2 ratio ≤13kPa/100 mmHg; *n* = 12). Further limitation is the lack of the measurement of respiratory mechanics before, during and after FFB.

## Conclusion

Frequently occurring haemodynamic and respiratory instability could be attributable to diagnostic FFB and BAL but no cases of cardiac rhythm disturbances, bleeding, pneumothorax or procedure related death were observed in the present study. The procedures should be conducted under careful supervision by experienced physicians. Only a randomized controlled trial that compares diagnostic FFB and BAL with a non-invasive strategy could ultimately establish the safety profile and clinical utility of these procedures in critically ill ventilated patients.
